# Non-communicable diseases surveillance: overview of magnitude and determinants in Kenya from STEPwise approach survey of 2015

**DOI:** 10.1186/s12889-018-6051-z

**Published:** 2018-11-07

**Authors:** Richard G Wamai, Andre Pascal Kengne, Naomi Levitt

**Affiliations:** 1 Integrated Initiative for Global Health, Department of Cultures, Societies and Global Studies, Northeastern University, Boston, MA USA; 2 Non Communicable Diseases Research Unit, South African Medical Research Council, Francie van Zijl Drive, Parow Valley, Cape Town, Western Cape South Africa; 3 Diabetic Medicine and Endocrinology, University of Cape Town, J47/86 Old Main Building Groote Schuur Hospital, Observatory, Cape Town, 7925 South Africa; 4 Chronic Disease Initiative for Africa (CDIA), University of Capetown, J47/86 Old Main Building Groote Schuur Hospital, Observatory, Cape Town, 7925 South Africa

## Background

Disease surveillance is a scientifically and legally established hallmark of population health whose goal is systematically collecting, interpreting and disseminating data to target and monitor interventions to reduce disease morbidity and mortality [1–5]. However, data is often either lacking or of low quality especially in low- and- middle-income countries (LMICs). For example, more than half of global deaths for 2015 did not have an established cause [6]. The Global Burden of Disease (GBD), the largest descriptive epidemiological study, show low rates of data quality for most LMICs during 1980–2016 [7]. Despite substantial improvements in data quality and cause of death establishment [8, 9], of over 50 health-related Sustainable Development Goals (SDGs) indicators identified in the World Health Statistics 2017 report, data is adequate for monitoring 36 indicators [10].

Lack of, and low, quality data is exacerbated by data inconsistencies between sources often due to different methodologies of data gathering, synthesis and reporting. For example, data from the GBD, differs significantly with data from the World Health Organization (WHO) [11, 12] and HIV estimates from national surveillance systems in high-income countries and UNAIDS [13]. In low-income countries (LICs) data from demographic and population surveys differ even within the same region for similar periods of time [14, 15]. Even when there are strategies in place like the Integrated Disease Surveillance and Response (IDSR) strategy for reporting communicable diseases in the WHO African region, they are poorly implemented [16, 17]. Discrepancies and gaps have led to a call for better data in recent years, e.g., by former WHO Secretary General [18], and subsequent development of Guidelines for Accurate and Transparent Health Estimates Reporting (GATHER) to define and promote best reporting practices for population studies in global and local contexts [19].

Without good data evidence-based public health decisions and interventions cannot be made [18, 20]. Current global burden of disease assessment shows multiple epidemiological transitions with rising non-communicable diseases (NCDs) and decreasing infectious diseases in prevalence, morbidity and mortality [6, 7]. In 2016, 54.7 million people died of which 72.3% were from NCDs and 8.4% from communicable, maternal, neonatal, and nutritional conditions (CMNN) [7]. Comprising 61.4% of 2.4 billion disability-adjusted life-years (DALYs) in 2016 [21], mortality from NCDs has risen 16.1% during 2006–2016 [7] and could reach 52 million by 2030 [22, 23]. On the other hand, mortality from all CMNN conditions including HIV/AIDS, tuberculosis, malaria and diarrheal diseases have declined over the same period by 24% overall [7]. Some LICs like Ethiopia have seen more dramatic decreases of up to 65% during 1990–2015 [24]. Although CMNN conditions remain a challenge, the decreases have resulted from key breakthroughs in prevention, treatment and control [25].

WHO data shows that 86% of deaths due to NCDs are in LMICs and most are premature [22]. Among these, cardiovascular diseases (CVDs), diabetes and cancer are the major killers [26]. With 8.2 million deaths and 14.1 million new cases in 2012, cancers are the single leading cause of world-wide deaths with 70% of the deaths occurring in LMICs [27, 28]. As the primary cause of CVD, hypertension is the leading risk factor for morbidity affecting 24% of adult men and 20% of adult women or 1.13 billion adults globally in 2015 [29]. Leading risk factors shared among the major NCDs – tobacco use, harmful consumption of alcohol, unhealthy diet, and physical inactivity – are all highly modifiable to prevent nearly half of premature NCD deaths [30–33].

Among LMIC regions, sub-Saharan Africa (SSA) faces the greatest challenge from NCDs that could pose the next ‘poverty trap’ [34]. While carrying the largest burden of major communicable diseases such as malaria, HIV/AIDS, tuberculosis, and neglected tropical diseases (NTDs) [35–38], the subcontinent is also undergoing critical epidemiological, demographic and socio-economic transformations including nutrition transitions that are resulting in a rise in NCDs such as cancer, hypertension, obesity and diabetes as earlier predicted [29, 39–44].An ageing and increasing population with simultaneously increasing number of people living with diseases like tuberculosis and HIV poses other challenges for NCD comorbidities [41, 44–49]. For example, a systematic review and meta-analytical study observed accelerated risk of pre-diabetes and diabetes among HIV-positive users of antiretroviral treatment (ART) due to ageing, dyslipidemia, metabolic syndrome, obesity and some ART medications that cause mitochondrial toxicity in long term use [50]. These effects have implications for the WHO recommendation to start ART treatment with initial HIV positivity test [51]. Owing to these patterns of disease, the case to invest more in health in Africa has been made by key global health agencies and key players on the continent including the WHO, the World Bank and the African Union [52].

Taking action on NCDs would save millions of lives and save LMICs an estimated $7 trillion in economic loses during 2011–2025 [22]. The growing prominence of NCDs led to the 2011 political declaration by the United Nations [53, 54]. Following this the WHO developed a Global Monitoring Framework for NCDs with nine voluntary targets to reduce mortality due to NCDs by 25% by 2025 [55], a target set to one third by 2030 in the SDG framework [56]. Towards this, surveillance data for NCDs are critically needed to inform interventions for the Global Action Plan for the Prevention and Control of NCDs 2013–2020 [32]. Recent reviews of approaches to data gathering for NCDs in LMICs find limited capacities and systems [3, 8, 57–59]. The approaches range from population and demographic surveys, vital registration systems, and socio-economic, behavioural and utilization surveys. Among the major approaches is the STEPwise approach to surveillance (STEPS) developed by the WHO in 2002 [60].

STEPS is a standardized NCD surveillance protocol involving three different levels of steps to gather self-reported data on demographics and behavioural risk factors physical and biochemical measures from nationally representative populations [60]. Since initiation the STEPS protocol has undergone multiple revisions given increasing body of knowledge and to serve the NCDs Global Monitoring Framework [61]. With over 120 countries implementing STEPS, it is the most widely used approach to collect data on NCDs and assess population risk-factor profiles to inform, monitor and evaluate policies and interventions [60, 61]. In the WHO African region 41 countries have implemented the STEPS. Here we summarize results of the Kenya STEPS appearing in this special journal supplement as captured in ten separate articles. We first overview the STEPS method followed by a summary of key findings in each article and then discuss these findings in context of their implications for NCDs surveillance and policy.

## Surveillance of NCDs through the STEPS in Kenya

Prior to the STEPS, national data on NCDs and related risk factors could have been estimated from the Kenya Demographic and Health Survey (KDHS), the 2014 Kenya Global Adult Tobacco Survey (GATS), the 2003 Global School Health Survey on physical inactivity and the GBD studies, and more recently the NCD Risk Factor Collaboration [29]. Covering numerous countries, the DHS Program started collecting data on NCDs by including a blood pressure module in 1998 [57]. In Kenya, the first DHS was conducted in 1989 but the NCD module was only added in the latest (2014) survey. The KDHS covers cancer and hypertension as well as three NCD risk factors (tobacco use, alcohol consumption, and physical inactivity), all of which are self-reported [62]. The GBD estimated that the contribution of NCDs to health loss (DALYs) amounted to 19.4% in 2000 rising to about 30% in 2013 [63]. Although the DHS uses a nationally representative sample and the GDB aggregates multiple data sources [57], these reports do not match the depth of measures, methodology and risk-factor analysis capabilities of the STEPS. Aside from the national DHS, NCD data for Kenya are also captured in rural-regional demographic surveys in western Kenya [64] and in the coastal region [65], and in the limited Nairobi cancer registry [66]. In addition, the first Global school-based student health survey (GSHS) was conducted in 2003 [67] and the first Global Tobacco Survey (GTS) in 2014 [68].

These and other disease-specific studies have provided knowledge of NCDs in Kenya as well as risk factors but the prevalence of diseases, their distribution and risk-factors at the national level have not been comprehensively examined. The DHSs have provided evidence that NCDs are prevalent not just in urban but also in rural areas and are rising [64, 65] while estimates based on the cancer registry indicate cancer contributes to 7% of national mortality [66, 69]. A unique study using the health production function estimated the effect of risk factors for NCDs based on national household expenditure and health utilization data [70]. Key risk factors to NCDs were socio-economic (income, education levels), health systems (e.g., distance to health facilities, cost of care), adverse social interactions in area of residence (e.g., availability and consumption of alcohol and smoking and influencing behavioural factors), and biological factors (e.g., age and gender) [70]. The STEPS has allowed a deeper and more comprehensive understanding of the national NCDs risk-factor profile.

### Overview of methods for the Kenya STEPS

The STEPs Kenya 2015 survey was a national cross-sectional household survey conducted between April and June 2015. It was designed to provide estimates for indicators on risk factors for NCDs for persons aged 18–69 years with a sample size of 6,000 individuals to allow national estimates by sex (male and female) and residence (urban and rural areas). Using a three-stage sampling, 200 clusters (100 urban and 100 rural) were selected in stage one, followed by a uniform selection of 30 households in each cluster in stage two, and in stage three one adult aged 18–69 years was randomly selected from each household, with a household defined as people who eat and live together, and approached for the survey. The survey used the fifth National Sample Surveys and Evaluation Programme (NASSEP V) master sample frame that is developed and maintained by Kenya National Bureau of Statistics. The stratified probability proportional to size sampling methodology was developed using 96,251 Enumeration Areas (EAs) generated from the 2009 Kenya Population and Housing Census to form 5,360 clusters split into four equal sub-samples [62]. National sampling frames are necessary for disease surveillance at the household level and evolve over time to reflect population and administrative changes [71]. The Kenya STEPS thus uses the most recent frame (NASSEP V) which was also used in the latest DHS.

The survey used the modular expanded STEPS collecting demographic and behavioural information (step 1), physical measurements (step 2) and biochemical measures (step 3) [60]. After providing informed consent, the participants were interviewed on the four main behavioural risk factors of NCDs (tobacco use, harmful use of alcohol, unhealthy diets, and physical inactivity), and measurements for key biological risk factors for NCDs (overweight and obesity derived from height and weight and central obesity derived from waist and hip circumference, blood pressure and fasting blood glucose, triglyceride, and cholesterol levels) were also taken. The survey was administered using a Personal Digital Assistant (PDA) loaded with the WHO eSTEPS software. Data collection took place during a two-month period (April–June) in 2015. Complete details of the sample design, methodology and questionnaire are provided in the formal report published by the Ministry of Health [72]. Each article also summarizes specific methodological method for the relevant question covered.

Research ethics approval for the Kenya STEPS was obtained from Kenya Medical Research Institute (SSC No. 2607). The survey was funded by the World Bank, but the Bank had no role in the decision to publish this special issue nor in interpretation of the results.

## Overview of the results of the Kenya STEPS

The ten individual articles covering the Kenya STEPS data reports the following main findings, without any specific order.

Mohamed and colleagues’ paper on hypertension focuses on the prevalence, awareness, treatment and control including determinants. The study reveals that the age-standardized prevalence for hypertension in Kenya was 24.5% which is similar to the global average [29] but lower than rest of sub-Saharan Africa at 30% [73]. Among those with hypertension 15.6% were aware of elevated blood pressure, of whom 26.9% were on treatment and 51.7% of those on treatment had achieved blood pressure control. Key factors associated with hypertension were older age, higher body mass index (BMI) and harmful use of alcohol, all of which are characteristic factors in other populations.

Another article by Mohamed and other colleagues analyses data on prevalence and factors associated with pre-diabetes and diabetes mellitus, finding the age-standardized prevalence at 3.1% and 2.4%, respectively. Nearly half (43.7%) were aware of having pre-diabetes or diabetes, a figure that is worse than the global estimate of undiagnosed diabetes of 50% but better than the Africa estimate of 69% [74]. About 20% were on treatment and only 7% of these had achieved glycaemic control. Low level of education was associated with lower odds of pre-diabetes while raised blood pressure, overweight/obesity among women, and older age (60–69 years) were associated with diabetes.

The article on cervical cancer looks at predictors of cervical cancer screening among women. Among 1,180 women interviewed only 16.4% had screened for cervical cancer despite high awareness (67.9% of unscreened women) of cervical cancer screening. Predictors of screening were higher education levels, being in the highest income quintile and living in urban areas.

Wekesah et al. analyze factors associated with the presence of multiple NCD risk factors at the individual and household levels. Key findings show that over two thirds (75.8%) of individuals in this study had four to six risk factors for NCDs and 10% had seven or more risks. Insufficient fruit and intake was almost universal (99.8%). The overwhelming majority (89.5% and 80.3%, respectively) consumed high dietary salt and had insufficient physical activity. The number of NCD risk factors increased with age among both men and women and household wealth index status. Associations of risks with other socio-demographic factors such as income-level, marital status, ethnicity and region differed between sexes. For example, married women had higher odds of more NCD risk factors.

Analysis of risk factors for NCDs and injury adds another perspective from the Kenya STEPS data. Using a K-median cluster analysis approach the article develops a risk profile of the population identifying five clusters for NCDs (hypertensives, harmful users, the hopefuls, the obese, and the fat lovers) and four clusters for injury (helmet users, jaywalkers, the defiant and the compliant). The strongest predictors of clustering were age, gender, education and wealth index. The article’s key message is that different portions of the population are exposed to differing risk factors creating the diverse risk profiles. Identifying these risk profiles is critical and confirms that policy formulation and interventions for injuries in particular and NCDs in general need to be differently targeted to mitigate exposure to risk factors, thus prioritizing a “best buys” approach.

The prevalence and the determinants of heavy episodic drinking (HED) were assessed by Kendagor and colleagues. Overall adult HED prevalence was determined to be 12.6% with men comprising 88.5% of this group. The 18–29 years old age group had the highest proportion (35.5%) of HED. HED was also high (60%) among the married/cohabiting but even higher among those who were separated. Home-brewed beers or wines were the most commonly reported by almost two thirds of the respondents. Consumption of tobacco was associated with higher odds of HED but the years of schooling, wealth quintile and residence did not have any effect on HED prevalence.

Levels and determinants of tobacco use is the focus of the article by Ngaruiya et al. Tobacco use was reported by 13.5% of the respondent sample of which 83.8% of users were men. Among the users 78.8% did so daily. The average age of starting tobacco use was 21 years and 75% of users were less than 50 years old and mostly in the 18–29 year-old age group. On the other hand, the 50–59 year age group were three times more likely to use tobacco daily than the younger age groups. Being male, adolescent/youth, and having lower level of education were the main determinants of tobacco use. Majority (71.7%) of users had no schooling beyond primary school. Tobacco smoking was more common in urban areas compared to rural areas where smokeless tobacco use was more predominant. Alcohol use had a moderate association on daily tobacco use. An insignificant number reported using e-cigarettes.

Assessing the prevalence and predictors of physical inactivity Gichu et al.’s study finds very low levels of physical inactivity with only 7.7% of respondents being classified as physically inactive. Being female and of middle age (30–49 years), and having higher education and income levels (middle wealth quintile) were the most important predictors of physical inactivity with area of residence (urban/rural) having little prediction effect.

Mwenda and co-authors analyse patterns of dietary risk factors and association with NCDs. Results show that high (unhealthy) intake of dietary salt and sugar was 18.3% and 13.7%, respectively. The majority of respondents were aware of health risk from dietary salt (88%) and sugar (91%) although just over half were implementing steps to reduce intake of salt and sugar (56% and 54% of respondents, respectively). Daily intake of a minimum of five servings of both fruits and vegetables was reported by only 6.0% the respondents. Male gender, being under 46 years old and being a student were strongly associated with unhealthy diet.

Gathecha and colleagues examine prevalence and predictors of injuries. Prevalence of injuries over past 12 months was 15.2% and 60.3% were males. The main causes of injuries were falls (47.6%), cuts (34.0%) and road traffic crashes (4%). Injuries due to violent incident was only reported by 3.7% of respondents. Injuries were significantly more prevalent among rural residents and 40.7% occurred at home, 16.9% in the farm and 16.7% in other work places. Consistent seatbelt use among drivers and passengers was reported by only 12.5%, 62% of motorcycle users never use helmets and drunk driving was reported by 8.1% of the drivers. Younger males age 18–29 years and smokers were significantly more likely to be injured in a road traffic accident. Binge drinking was a factor in violent incidents as was age 30–39 years. Being in the top two income quintiles was protective against unintentional injuries.

## Discussion

These studies from the Kenya STEPS provide the most comprehensive understanding of NCDs and associated risk factors in the country. To a large degree the results reflect the current stage of demographic and economic transitions. Overall, according to data from the KDHS deaths due to NCDs increased from 35% of total deaths in 2003 to 45% in 2010 [64]. In 2018, Kenya’s population is estimated at 50.9 million with 72.9% living in rural areas [48]. After dipping due to the HIV/AIDS crisis in the 1990s, life expectancy has been increasing since 2000 as transitions continue to occur in health and the economy with a growing urbanization and an expanding middle class [41].

Correspondingly, levels of physical inactivity, overweight/obesity nutrition transitions reflect population distribution and economic activity. For example, physical inactivity in the STEPS was only 7.7% compared with that in other higher income-level countries like South Africa (44.7%) and Swaziland (49.1%) and the global prevalence of 31% [75]. Without strong culturally mitigating effects, disease transition models predict these developments to continue to create a society where NCDs dominate commensurate with the dominant risk factor environment [39, 41, 76–79]. For that reason, Kenya needs to vigorously take measures to meet the goals laid out in the Global Action Plan for the Prevention and Control of NCDs 2013–2020 [32], the country’s Vision 2030 for a healthy and prosperous nation [80], and the SDG #3 target 4 of reducing premature mortality due to NCDs by one third by 2030 [81].

The health effects of NCDs and related risk-factors are well established [33, 82–86]. As the Kenya STEPS data show, socio-economic and demographic factors namely, income level, age, gender, urban residence and education are associated with NCDs [70, 72]. Taking action on NCDs would save millions of lives and economic productivity [34, 87, 88]. The WHO estimated that just $11.2 billion in annual investments could save $7 trillion in lost productivity in LMICs during 2011–2025 [22]. As such, interventions for NCDs are highly cost-effective. For example, nutrition education in New York City public schools was cost-effective in reducing childhood obesity [89]. Effective policy changes are also cost-effective and will decrease NCD costs and prevalence making it the best way to improve overall health for all [90]. Addressing factors contributing to excessive weight gain; smoking and treating hypertension would be cost-effective in tackling NCDs in Kenya [41].

Health benefits resulting from reducing risks for NCDs are huge. Increased longevity resulting from positive change in lifestyle is evident even in older adults aged over 50 years with or without CVDs [83]. Large nationally representative data in the US has recently shown that, compared to the status quo, maintaining a healthy BMI, eating a healthy diet, a moderate use of alcohol, and moderate to vigorous exercise could add over a decade of life for men and women over 50 years of age [85]. Interventions to target one risk factor can often have cascading effects. For example, because physical inactivity is shown to contribute to numerous NCDs including cancer and approximately 30% of ischaemic heart disease [84] programs to increase physical activity could have huge effects.

Kenya has a national strategy for prevention and control of NCDs for 2015–2020 [91]. A new cancer control strategy for 2017–2022 was recently launched [92]. In addition, the country also has strong policies on smoking and alcohol, namely the Framework Convention for Tobacco Control (FCTC) which the country ratified in 2004, the Kenya Tobacco Control Act of 2007 and the 2010 Alcoholics Drinks Control Act. Despite these policies, laws and strategies, capacity to document, implement, monitor, and evaluate NCD programming in the country as in other LMICs is woefully inadequate [57, 58]. In the 2014 WHO NCDs Country Profiles, Kenya reported negative to having any of the nine national systems to respond to NCDs [93]. The latest (2013) *Kenya Service Availability and Readiness Assessment Mapping (SARAM)* showed that overall only 5% of facilities offered all NCD services defined in the Kenya Essential Package of Health (KEPH) and only 25% of health facilities had different tracer commodities for NCDs with huge regional variations [94]. Strengthening core data for NCDs in vital registration and integrating risk-factor surveillance into policy and programming is essential [59]. Because of complex challenges in standardization experts recommend utilization of mixed data systems, a practice currently in place everywhere although some countries have more robust structures than others [3, 57, 59].

This special journal issue of the Kenya STEPs survey brought numerous co-author teams together drawn from the Kenya Ministry of Health, the African Population and Health Research Centre (APHRC), other entities and academia both locally and abroad. Altogether, 35 comprising scholars and practitioners in many disciplinary fields contributed to the special issue. The 10 research articles were peer-reviewed by a team of 22 reviewers and 3 editors from different disciplines and institutions around the world. The publication demonstrates the value for international collaborations in research on NCDs. Such collaborations provide opportunities but also challenges [95]. In our case, inter-institutional and interdisciplinary north-south and south-south collaborations were key strengths. Such collaborations provide benefits for science, academic and implementing institutions, and for the individual investigators. In an era where team research is increasingly promoted [96] true collaborations could help improve equity in international health research and increase output by African scholars and practitioners [97, 98]. To our knowledge this is the first time any country has organized to publish comprehensive STEPS data in a special issue in the peer-reviewed literature.

## Conclusions

The 2015 Kenyan STEPS survey provides the first snapshot of major NCDs risk factors burden in the country, serving as baseline against which future progresses will be monitored. Such monitoring should include a repetition of the survey at regular time-intervals. The scope of NCDs and risk factors is too broad to be comprehensively covered in a single survey, including in high-income countries. However, by strategically building into successive surveys, specific modules for key NCDs/risk factors, countries over time are able to generate surveillance data for major established and emerging NCDs in their population. Kenya could use a similar approach in future STEPS or similar surveys, to expand the scope of traditional NCD risk factors to lipid disorders including expanding the assessment of diabetes beyond a fasting glucose level that is likely to underestimate the prevalence, to collect data on neglected NCDs like epilepsy and mental health, and on emerging and novel risk factors. This should ideally go in parallel with efforts to develop and maintain complementary data sources, which in the long term, will equip the country with a comprehensive surveillance system for NCDs. With such a pragmatic policy prescription for NCD surveillance, the country also need to step up provision of services for prevention and treatment of NCDs in and outside the health system in line with national and global goals. Ultimately, for Kenya and countries across Africa the path to longer and healthier lives to 2030 [99], and beyond, requires understanding disease profiles matched by the right interventions.

## Abbreviations

APHRC: African Population and Health Research Centre
ART: antiretroviral treatment
BMI: Body mass index
CDIA: Chronic Disease Initiative for Africa
CHANCES: Consortium on Health and Ageing Network of Cohorts in Europe and the United States
CMNN: communicable, maternal, neonatal, and nutritional conditions
CVD: Cardiovascular diseases
DALYs: disability-adjusted life-years
DHS: Demographic and Health Survey
EAs: Enumeration Areas
FCTC: Framework Convention for Tobacco Control
GATHER: Guidelines for Accurate and Transparent Health Estimates Reporting
GATS: Global Adult Tobacco Survey
GBD: Global Burden of Disease
GSHS: Global school-based student health survey
GTS: Global Tobacco Survey
HALE: healthy life expectancy
HDL: High-density lipoproteins
HDSS: Health and Demographic Surveillance System
HED: heavy episodic drinking
IDSR: Integrated Disease Surveillance and Response
KDHS: Kenya Demographic and Health Survey
KEPH: Kenya Essential Package of Health
KNBS: Kenya National Bureau of Statistics
LICs: low-income countries
LMICS: Low- and middle-income countries
NASSEP V: National Sample Surveys and Evaluation Programme
NCDs: Non-communicable diseases
NTDs: neglected tropical diseases
PCA: principal component analysis
PDA: Personal digital assistant
SARAM: Kenya Service Availability and Readiness Assessment Mapping
SDGs: Sustainable Development Goals
SSA: Sub-Sharan Africa
STEPS: WHO STEPwise approach to Surveillance (of NCD risk factors)
WHO: World Health Organization
